# Is it possible to bridge the Biopsychosocial and Biomedical models?

**DOI:** 10.1186/1751-0759-8-3

**Published:** 2014-01-14

**Authors:** Richard D Lane

**Affiliations:** 1 Department of Psychiatry, University of Arizona, Tucson, AZ, USA

## 

It is an honor to be invited to offer a perspective on our field for the official journal of the Japanese Society of Psychosomatic Medicine. As a past leader of the American Psychosomatic Society, I view this as an opportunity to continue building bridges regarding our common interests. One of the challenges of effective and meaningful communication in this context is being mindful of our similarities and differences. Awareness of differences can help to overcome communication challenges within our own field as well as when we seek to communicate and collaborate with our colleagues in other medical disciplines.

To some of us it is obvious that biological, psychological and social factors are in reality integrated and that biopsychosocial medicine seeks to elucidate this reality. The biomedical, organ-based perspective focuses on disease mechanisms and assumes that the psychological and social are not essential to understanding and treating patients, although humanism in patient care is of course endorsed. Bridging these perspectives is important because the biomedical is the predominant model adopted by those who decide how to allocate health care dollars in the United States and many other countries. This in turn determines what clinical care is provided at the bedside.

At the September 2013 meeting of the International College of Psychosomatic Medicine in Lisbon a tribute to George Engel was held. The symposium highlighted the value of the biopsychosocial model that he created and highlighted the limitations of the prevailing biomedical model. The predominant messages of the symposium appeared to be that the biopsychosocial approach had many advantages, it did not exclude the biomedical perspective, and that our biomedically-oriented colleagues should join us.

The unfortunate reality is that in the 37 years since Engel’s seminal paper in Science [1] was published the integration of the biopsychosocial approach into everyday medical care hasn’t happened yet, and there is no sign in the foreseeable future that it will. With health care costs escalating there is less and less time for physician-patient contact. Medical encounters and reimbursements are structured to cover the essentials of the biomedical approach. The comprehensive nature of the biopsychosocial approach to clinical care appears to be a luxury that we can’t afford. To change the funding climate we need to somehow convey our message to key decision-makers more effectively.

I submit that in order to do so we need to speak to our biomedically-oriented colleagues in their language. We need to find ways to explain the biopsychosocial model in biomedical terms, i.e. specific quantifiable mechanisms that demonstrate a causal chain of events. Although this will not capture all of the complexity and richness of the biopsychosocial model and interactions across levels, it does provide a bridge between two fundamentally different perspectives.

Neuroscience will be an essential ingredient in this undertaking. Psychological and social phenomena are mediated by the brain. Since the brain is in the body, the mechanisms of brain-body interaction will be more readily understandable than mind-body interactions. This in no way minimizes the value of studying psychological and social factors in their own right, as our understanding of the neural basis of psychological and social factors is in its infancy. For the purpose of communicating with policy-makers and funders, and ultimately improving health care, our empirical understanding of the role of the brain in mind-body interactions needs to be expanded. Adding to the challenge is that most biopsychosocial clinicians and researchers are not neuroscientists. Collaborations with neuroscientists will therefore be essential. Two white papers have been written to facilitate such collaborations [2,3], and a Special Issue of Neuroimage demonstrated the potential of Brain-Body Medicine research [4].

Within this context the concept of mentalizing may play a particularly important organizing role in bridge-building because it provides a focal point for integrating psychological, social, brain and peripheral physiological phenomena. Mentalizing is fundamentally the cognitive skill of understanding why people do what they do. This skill involves determining the mental state that explains behavior [5]. This skill applies both to the self and others and applies to thought as well as emotion [6]. The ability to appreciate what others are thinking and feeling is critical to making, maintaining and enhancing social relationships.

From a psychological perspective mentalizing includes self-awareness. Knowing what you are feeling is a pre-requisite for intentional emotion self-regulation. Therapies such as mentalization-based therapy [7], emotion-focused therapy [8] and cognitive-behavioral therapy [9] all benefit from and seek to promote self-awareness. In the Eastern tradition meditation is an ancient method that promotes self-awareness and resilience. Effective social interaction requires integrating the perspectives of self and other in the immediate context. One way of understanding and reconciling the cross-cultural perspectives of West and East is to appreciate their relative focus on the individual and the social context, respectively [10]. Such differences have been linked to how the medial prefrontal cortex is engaged during mentalizing tasks [11].

The brain basis of mentalizing is increasingly being elucidated. The medial prefrontal cortex is a central hub connected to the temporo-parietal junction and anterior temporal cortex in the service of interpreting biological motion such as facial expressions and gestures and recalling previous experiences in the service of interpreting the current context. The medial prefrontal cortex is also a key node for self-monitoring and self-regulation [12]. For example, the subgenual, pregenual and dorsal anterior cingulate cortices are key nodes in autonomic and visceral regulation through their connections with each other and brainstem effector regions [2]. The medial prefrontal cortex is therefore a key node for understanding the brain basis of the influence of psychological and social factors on bodily processes.

The medial prefrontal cortex has also been shown to play a key role in the regulation of vagal tone [13]. Vagal tone is mediated by a hierarchy of neural structures with the medial prefrontal cortex sitting atop the hierarchy. Vagal mechanisms have either direct or indirect effects through the sympathetic nervous system on all bodily organ systems. When it is considered that vagal stimulation is anti-inflammatory [14], the range of effects on health and disease multiply further. The ultimate effect of vagal influences can be understood within the wider context of the relative balance between the sympathetic and parasympathetic nervous systems, or autonomic balance, which, like vagal tone, is regulated by the brain [15].

One can therefore envision a causal chain in which social events, psychological processes, brain mechanisms, autonomic, neuroendocrine, immune mediators and organ-specific disease mechanisms are linked through specifiable biological mechanisms. To the extent that we can elucidate such causal pathways through additional research it will make the processes described by the biopsychosocial model understandable to our biomedical colleagues. This will be especially true if we can develop intervention methods that influence function at different levels of organization and demonstrate the mechanisms of cross-level effects. Addressing how the processes at work change as a function of medical condition or cultural context will further extend the model.

Mentalizing is but one example of a focus that can lead to elucidation of the mechanisms linking the different levels of the biopsychosocial model. Such a mechanistic model, which might be considered an illustration of the biomedical approach, can potentially facilitate understanding of the strengths and limitations of each perspective. Facilitating this type of communication through new research and creation of a comprehensive model such as this may be considered an example of the biomedical perspective contributing to the betterment of the biopsychosocial approach. Perhaps the beneficial effect of such bridge-building will be a two-way street if it also leads to an expansion of the biomedical perspective.
